# Coapplication of Adenine with Inosine or Guanosine Supports Rapid ATP Restoration by ATP-deprived Cultured Primary Astrocytes

**DOI:** 10.1007/s11064-025-04511-x

**Published:** 2025-08-23

**Authors:** Gabriele Karger, Ralf Dringen

**Affiliations:** 1 Centre for Biomolecular Interactions Bremen, Faculty 2 (Biology/Chemistry), University of Bremen, P.O. Box 330440, 28334 Bremen, Germany; 2 Centre for Environmental Research and Sustainable Technologies, University of Bremen, Bremen, Germany

**Keywords:** Astrocytes, ATP restoration, Guanosine, Inosine, Purine nucleosides, Purine salvage pathway, Ribose

## Abstract

Astrocytes contain a high concentration of adenosine triphosphate (ATP) that enables these cells to perform their physiological functions in brain. To investigate the mechanisms involved in astrocytic ATP restoration, the ATP content of cultured primary rat astrocytes was first depleted by a preincubation with the mitochondrial uncoupler BAM15 before extracellular substrates and their combinations were applied to foster ATP restoration. To test for the contribution of the purine salvage pathway to synthesize new adenosine monophosphate (AMP) for ATP restoration, several purine nucleosides and purine bases as well as their combinations were applied. In the absence of glucose, partial ATP restoration was found for incubations with inosine and guanosine that was lowered by forodesine, an inhibitor of purine nucleoside phosphorylase. In glucose-fed cells, the coapplication of micromolar concentrations of adenine with inosine or guanosine, but not with ribose, accelerated ATP restoration in a concentration-dependent manner. By such treatments, 80% of the initial ATP content were restored within 40 min. The supporting effects of inosine and guanosine on ATP restoration were prevented by the presence of forodesine, demonstrating the contribution of purine nucleoside phosphorylase in the ATP restoration observed. These data demonstrate that ATP-deprived astrocytes need for rapid ATP restoration - in addition to glucose as energy substrate - an adenine source and inosine or guanosine as precursor for the ribose phosphate moiety of ATP.

## Introduction

Astrocytes represent a substantial part of the cells in the brain and have a variety of important functions as partners of neurons [1–3]. For example, astrocytes are involved in intercellular processes such as signaling and neurotransmitter recycling [4, 5], handling of reactive oxygen species [6–8] and managing the brain´s energy metabolism [9–12]. To fuel their energy demand astrocytes contain a millimolar concentration of adenosine triphosphate (ATP) [13, 14] that is efficiently and continuously regenerated by cytosolic glycolysis and mitochondrial oxidative phosphorylation [13–19].

Severe impairment of ATP regeneration causes a transient increase in cellular contents of adenosine diphosphate (ADP) and adenosine monophosphate (AMP), but the overall content of adenosine phosphates declines under such conditions most likely by further metabolism of AMP [14, 17]. For restoration of a normal high ATP content after such treatments, synthesis of new AMP is required that is subsequently phosphorylated via ADP to finally restore ATP [17]. Two cellular pathways are known to contribute to the synthesis of new AMP, the purine d*e novo* synthesis and the purine salvage pathway [20, 21–25]. For brain cells, the *de novo* synthesis of purine bases from amino acids precursors is discussed to play only a minor role, while the purine salvage pathway appears to be rather active [24, 25]. By the salvage pathway, preexisting purine bases such as adenine and hypoxanthine are transferred by phosphoribosyltransferases (PRT) onto phosphoribosyl pyrophosphate (PRPP) to generate AMP and inosine monophosphate (IMP), respectively [25, 26]. The latter can then by amidated to AMP [25, 27].

Astrocytes are well known to metabolize exogenous purine nucleosides [17, 28–30]. Such nucleosides can be taken up by equilibrative nucleoside transporters [30–36] into astrocytes [16, 25, 37, 38]. In the cells, the enzyme purine nucleoside phosphorylase (PNP) removes the base from the nucleoside and generates ribose-1-phosphate [21, 25, 39] that can subsequently be isomerized to ribose 5-phosphate, an intermediate of the pentose-phosphate pathway (PPP). This phosphorolytic cleavage of nucleosides is likely to be involved in their utilization as exogenous substrates that help to maintain a high cellular ATP content in starved astrocytes [16, 40]. In addition, adenosine in micromolar concentrations can serve as building block to foster rapid ATP restoration in ATP-depleted astrocytes [17]. However, under physiological conditions adenosine is present only in nanomolar extracellular concentrations in brain [41] and this nucleoside acts mainly as extracellular signaling molecule [42, 43].

We have recently reported [17] that cultured astrocytes that had been depleted of most of their ATP by a preincubations with the mitochondrial uncoupler BAM15 in the absence of glucose are only slowly and partially restoring ATP after refeeding energy substrates. However, this process can be strongly accelerated by a coapplication of adenosine, suggesting that both an adenine sources as well as an energy source are needed for efficient ATP restoration in ATP-depleted astrocytes [17]. After uptake, adenosine is efficiently phosphorylated in astrocytes to AMP [17], which is further phosphorylated to ATP. However, as extracellular adenosine is highly neuroactive and has many signaling functions in brain [42–44], a potential application of this nucleoside as precursor for ATP restoration in vivo has to be excluded [24].

To bypass this adenosine problem, we have extended our initial study on the ATP restoration in ATP-deprived cultured astrocytes by investigating the potential of astrocytes to use free purine bases and other purine nucleosides as precursors for ATP restoration. Here we report that the slow glucose-dependent ATP restoration by ATP-depleted astrocytes was only slightly improved by application of adenine, inosine or guanosine, but substantially accelerated by coapplication of adenine plus either inosine or guanosine. The supporting effect of the purine nucleosides was prevented by an inhibitor of purine nucleoside phosphorylase, suggesting that the purine nucleosides deliver additional ribose phosphate that is required for efficient synthesis of new AMP in astrocytes by the salvage pathway. These data demonstrate that the application of a combination of glucose as energy substrate with adenine as precursor of the adenine moiety of ATP and with either inosine or guanosine as precursor of the ribose-phosphate moiety enables ATP deprived cultured astrocytes to rapidly restore ATP.

## Materials and methods

### Materials

Sterile cell culture consumables, unsterile 96-well plates and black microtiter plates were purchased from Sarstedt (Nümbrecht, Germany). Sodium acetate, bovine serum albumin, dimethyl sulfoxide (DMSO), NAD^+^ and NADPH were obtained from AppliChem (Darmstadt, Germany). Adenosine triphosphate (ATP), glutamate-pyruvate transaminase, lactate dehydrogenase (LDH) and pyruvate kinase were purchased from Roche Diagnostics (Mannheim, Germany). Adenine, adenosine, guanosine, hypoxanthine, inosine, AMP, BAM15 and glucose-free Dulbecco’s modified Eagles medium (DMEM) were obtained from Sigma-Aldrich (Steinheim, Germany). Fetal calf serum (FCS) and penicillin G/streptomycin sulfate solution were from Thermo Fisher Scientific (Schwerte, Germany). Forodesine hydrochloride was obtained from MedChemExpress (Monmouth Junction, NJ, USA). The Cell Titer Glo^®^ 2.0 ATP Assay Kit was from Promega (Walldorf, Germany) and UK5099 from Merck (Darmstadt, Germany). All other basal chemicals were obtained from Sigma-Aldrich (Steinheim, Germany), Roth (Karlsruhe, Germany) or Merck (Darmstadt, Germany).

### Astrocyte-rich Primary Cultures

Primary astrocyte-rich cultures were prepared from the brains of newborn Wistar rats as previously described in detail [45]. The rats were purchased from Charles River Laboratories (Sulzfeld, Germany) and treated in accordance to the State of Bremen, German and European animal welfare acts. The harvested cells (1 mL) were seeded in a density of 300,000 viable cells per milliliter culture medium (90% DMEM containing 25 mM glucose, 44.6 mM sodium bicarbonate, 1 mM pyruvate, 20 U/mL penicillin G, 20 µg/mL streptomycin sulfate, supplemented with 10% FCS) in wells of 24-well dishes. The cultures were maintained in the humidified atmosphere of a Sanyo (Osaka, Japan) CO_2_ incubator containing 10% CO_2_. The culture medium was renewed every seventh day and one day prior to an experiment. For experiments, confluent primary astrocyte cultures of an age between 14 and 31 days after seeding were used. Astrocyte-rich primary cultures are strongly enriched in astrocytes and contain only low numbers of contaminating other glia cell types [45–47].

### Experimental Incubation of Cultured Astrocytes

Confluent astrocytes in wells of 24-well dishes were washed twice with 1 mL pre-warmed (37 °C) glucose-free incubation buffer (IB; 145 mM NaCl, 20 mM HEPES, 5.4 mM KCl, 1.8 mM CaCl_2_, 1 mM MgCl_2_, 0.8 mM Na_2_HPO_4_, pH adjusted with 5 mM NaOH to 7.4 at 37 °C) and subsequently incubated for 60 min in 250 µL IB containing 1 µM of the uncoupler BAM15 at 37 °C in the humidified atmosphere of a CO_2_-free incubator to lower the cellular ATP concentration. To study ATP restoration, the cells were subsequently washed twice with 1 mL pre-warmed (37 °C) glucose-free IB to remove the uncoupler and incubated for ATP restoration in 250 µL IB containing substrates and/or inhibitors as indicated in the legends of the figures for up to 6 h in the humidified atmosphere of a CO_2_-free incubator. After the given incubation periods, the media were harvested from the cultures to test for cell viability by measuring the extracellular activity of lactate dehydrogenase (LDH). For ATP measurements, the cells were washed twice with 1 mL ice-cold (4 °C) phosphate-buffered saline (PBS; 10 mM potassium phosphate buffer pH 7.4 containing 150 mM NaCl) and lysed as described below.

### Determination of Cellular Contents of ATP

To measure the cellular contents of ATP, the washed cells were lysed in 200 µL of ice-cold 0.5 M HClO_4_ on ice for 5 min and the collected lysates were neutralized by adding a predetermined specific volume of 2 M KOH. ATP in the neutralized lysates was determined as recently described [15–17] by a luciferine-luciferase-based luminometric assay using the Cell Titer Glo^®^ 2.0 ATP Assay Kit. For the calculation of the specific cellular ATP content, the determined ATP values were normalized on the protein content of the respective culture.

### Determination of Extracellular Lactate

Extracellular lactate that had been released from the cells during the incubation was determined by a coupled enzymatic assay with LDH and glutamate-pyruvate transaminase as previously described [45]. The absorption at 340 nm of the NADH generated during the enzymatic reaction was used to calculate the extracellular lactate concentration [45].

### Determination of Cellular Protein Content and Cell Viability

The protein content of astrocyte cultures was determined by the Lowry method [48] using bovine serum albumin as standard protein. To test for potential toxicity of a given treatment, the extracellular activity of LDH was measured in 10 µL media samples and compared with the total initial LDH activity in the respective culture as determined for Triton X-100 lysates [45]. None of the conditions investigated in the current study caused any significant increase in the extracellular LDH activity compared with the respective control condition.

### Data Presentation and Statistical Analysis

The data shown in the figures represent means ± standard deviations (SDs) of values obtained in n independent experiments that had been performed in duplicates on the given number of independently prepared astrocyte primary cultures. Basal cellular parameters from the experiments performed to obtain the data which are presented in the figures as well as the number of independently performed experiments (n) are summarized in Table 1. Normal distribution was confirmed by the Kolmogorov-Smirnov test for the data shown in Fig. 5 (*n* = 6) and Table 1 (*n* = 24), while normal distribution was assumed for all data sets derived from less than 5 independent experiments. Analysis for statistical significance between multiple groups of data was performed by ANOVA and the Bonferroni post-hoc test. Differences between two data groups were analyzed for statistical significance by the paired t-test. The calculated levels of significance compared to the respective control conditions are given in the figures as stated in the respective legends. *p* > 0.05 is considered as not significant.

**Table 1:** Basal cellular parameters obtained for the cell culture experiments performed to obtain the data that are presented in the figures of this Article

	Initial ATP content	ATP content after 60 min BAM15	Protein content	Initial LDH activity	*n*
	(nmol/mg)	(nmol/mg)	(µg/well)	nmol/(min x well)	
Figure 1	32.8 ± 2.1	8.2 ± 1.8	119 ± 8	111 ± 5	3
Figure 2	32.5 ± 1.2	8.0 ± 2.1	133 ± 14	115 ± 15	3
Figure 3a	29.6 ± 2.1	11.0 ± 2.8	135 ± 36	122 ± 35	3
Figure 3b	34.9 ± 4.0	6.1 ± 1.0	127 ± 2	101 ± 9	3
Figure 4	29.8 ± 2.5	8.7 ± 1.7	146 ± 20	144 ± 53	3
Figure 5	32.2 ± 1.7	7.5 ± 1.7	128 ± 13	112 ± 31	6
Figure 6	32.2 ± 1.2	6.7 ± 3.2	147 ± 25	105 ± 20	3
Figure 7	28.2 ± 1.0	5.8 ± 1.3	140 ± 14	105 ± 22	3
Figure 8	29.9 ± 3.4	7.8 ± 2.2	152 ± 24	118 ± 19	3
Average	31.3 ± 3.0	7.8 ± 2.4	133 ± 20	115 ± 29	24

Shown are the initial specific contents of ATP, the protein contents, the total cellular LDH activities as well as the ATP contents determined after a 60 min preincubation with BAM15 to lower ATP content before the main incubation for ATP restoration was performed. In addition, the number of independent experiments (n) that were performed on independently prepared cultures is given. The given average values are derived from a total of 24 independent experiments performed on 21 independent cultures. This number is lower than the sum of the experiments used to obtain the data shown in the figures of this article, as data derived from different experiments that had been performed on the same culture on a given day were considered only once for the calculation of the average values

## Results

### Test for Purine Bases and Nucleosides as Substrates for ATP Restoration in ATP-depleted Astrocytes

Cultured astrocytes that had been partially depleted of ATP by a 60 min preincubation with the uncoupler BAM15 in glucose-free buffer are able to fully restore their initial ATP content after application of suitable ATP precursors [17]. This experimental paradigm was used to study the potential of cultured astrocytes to use a variety of compounds as substrates and precursors for ATP restoration. Basal cellular parameters for the experiments performed to obtain the data which are shown in the Figures, such as the initial contents of ATP and protein, the initial LDH activities and the ATP content after the 60 min preincubation with BAM15, are listed in Table 1. The 60 min preincubation with BAM15 in glucose-free buffer lowered the cellular ATP content on average to 25% ± 7% (Table 1), consistent with literature data [17].

**Fig. 1:**
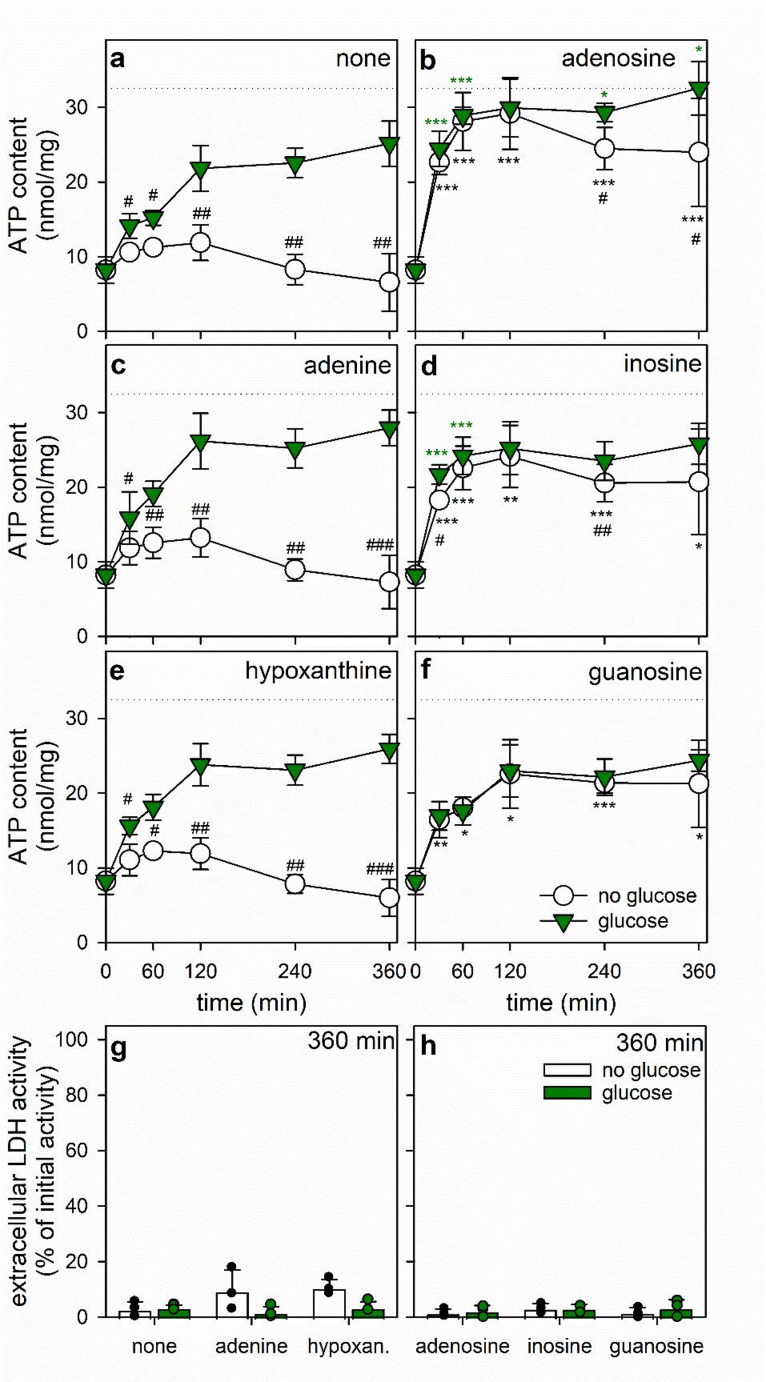
Time-dependency of the ATP restoration by ATP-deprived cultured astrocytes in the presence of purine bases or nucleosides. The cells had been preincubated for 60 min in glucose-free IB with 1 µM of BAM15 to lower the cellular ATP content before the cells were incubated without or with 5 mM glucose in the absence (**a**, **g**) or the presence of 1 mM adenosine (**b**, **h**), adenine (**c**, **g**), inosine (**d**, **h**), hypoxanthine (hypoxan.; **e**, **g**), or guanosine (**f**, **h**). The cellular ATP contents were determined for the given incubation periods (**a–f**) and the extracellular LDH activities after the 360 min incubation (**g**, **h**). The initial specific ATP content (Table 1) is indicated by the dotted lines in panels **a–f**. The significance of differences (t-test; *n* = 3) between data obtained for glucose-free and glucose-containing incubations is indicated by ^#^*p* < 0.05, ^##^*p* < 0.01 and ^###^*p* < 0.001. The significance of differences (ANOVA; *n* = 3) of data compared to the respective control incubation (none, panel a, absence of any purines or nucleosides) is indicated by *p < 0.05, **p < 0.01 and ***p < 0.001 in the colors of the respective symbols indicating the values for incubations without or with glucose

**Fig. 2:**
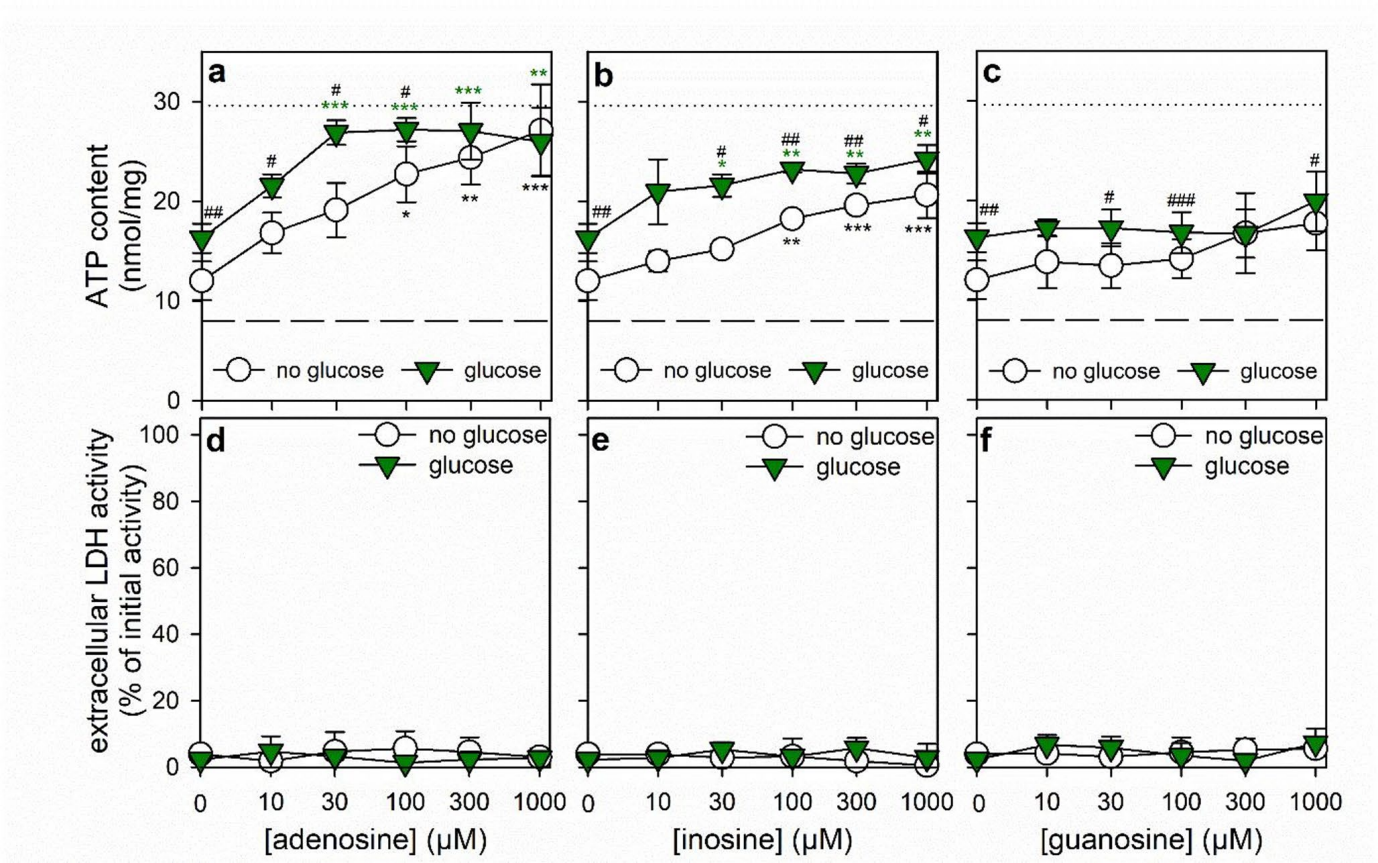
Dependency of the ATP restoration by ATP-deprived cultured astrocytes on the concentration of nucleosides applied. The cells had been preincubated for 60 min in glucose-free IB with 1 µM of BAM15 to lower the cellular ATP content before the cells were incubated without or with 5 mM glucose in the presence of the given concentrations of adenosine (**a**, **d**), inosine (**b**, **e**) or guanosine (**c**, **f**). After 60 min main incubation, the cellular ATP contents (**a–c**) and the extracellular LDH activities (**d–f**) were determined. In panels a-c, the initial ATP content of the cultures and the ATP content determined after the 60 min preincubation (Table 1) are indicated by the black dotted lines and the dashed lines, respectively. The significance of differences (ANOVA; *n* = 3) compared with the data obtained for the control incubation (absence of nucleosides) is indicated by *p < 0.05, **p < 0.01 and ***p < 0.001. The significance of differences (t-test; *n* = 3) between data obtained for glucose-free and glucose-containing incubations is indicated by ^#^*p* < 0.05, ^##^*p* < 0.01 and ^###^*p* < 0.001

**Fig. 3:**
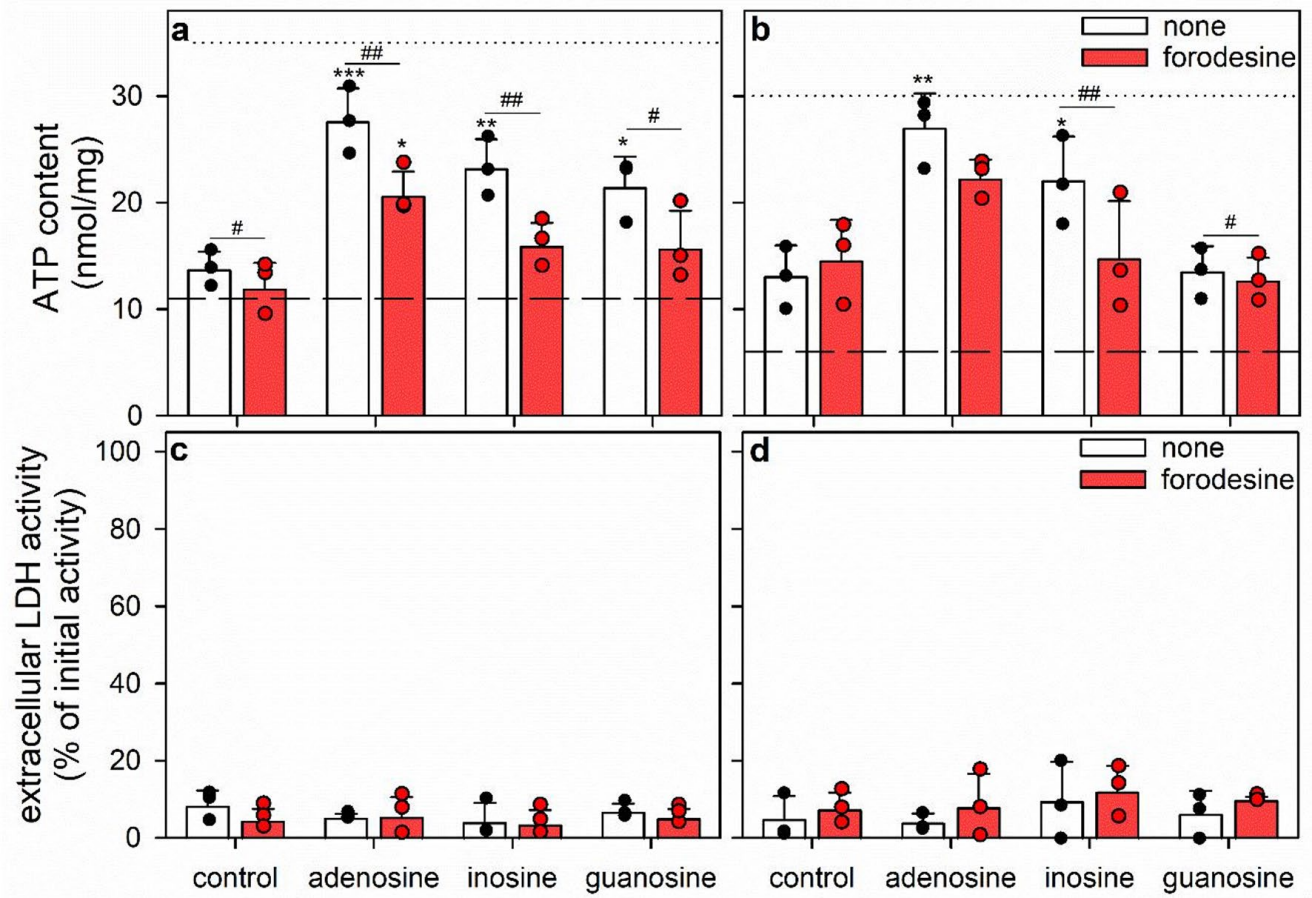
Effects of the PNP inhibitor forodesine on the use of nucleosides for ATP restoration by ATP-depleted astrocytes. Astrocyte cultures had been preincubated for 60 min in glucose-free IB with 1 µM of BAM15 to lower the cellular ATP content before the cells were incubated for 60 min in IB either without glucose in the absence or the presence of 1 mM nucleosides (**a**, **c**) or with 5 mM glucose in the absence or the presence of 30 µM nucleosides (**b**, **d**). Incubations were done without and with the PNP inhibitor forodesine (10 µM). After 60 min, the cellular ATP contents (**a**, **b**) and the extracellular LDH activities (**c**, **d**) were determined. In panels a and b, the initial ATP contents of the cultures and the ATP contents determined after the 60 min preincubation (Table 1) are indicated by the black dotted lines and the dashed lines, respectively. The significance of differences (ANOVA; *n* = 3) compared with the data obtained for the nucleoside-free control incubation is indicated by ^*^p < 0.05, ^**^p < 0.01 and ^***^p < 0.001. The significance of differences (t-test; *n* = 3) between data obtained for forodesine-free (none) and forodesine-containing incubations is indicated by ^#^*p* < 0.05 and ^##^*p* < 0.01

**Fig. 4:**
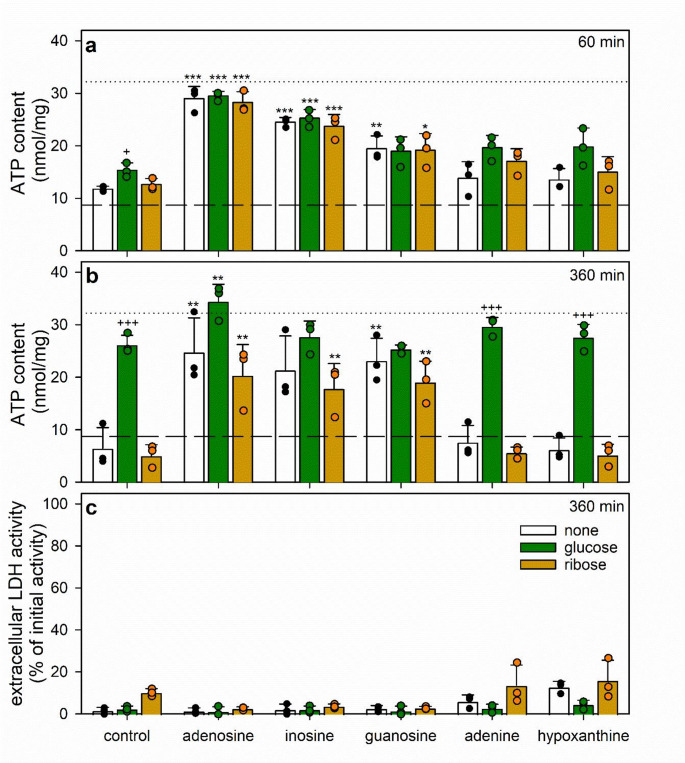
ATP restoration by ATP-deprived astrocytes in the absence or the presence of glucose, ribose, purine bases and/or purine nucleosides. The cells had been preincubated for 60 min in glucose-free IB with 1 µM of BAM15 to lower the cellular ATP content before the cells were incubated without or with 5 mM glucose or 5 mM ribose in the absence (control) or the presence of 1 mM of adenine, hypoxanthine, adenosine, inosine or guanosine, for 60 min (**a**) or 360 min (**b**, **c**). The cellular ATP contents were determined for the 60 min and 360 min time points and the extracellular LDH activities for the 360 min incubations. In panels a and b, the initial ATP content of the cultures and the ATP content determined after the 60 min preincubation (Table 1) are indicated by the black dotted lines and the dashed lines, respectively. The significance of differences (ANOVA; *n* = 3) compared with the data obtained for the respective control incubation is indicated by *p < 0.05, **p < 0.01 and ***p < 0.001. The significance of differences (ANOVA; *n* = 3) compared with the ATP data obtained without glucose and ribose (none) in the presence of the stated purines is indicated by ^+^*p* < 0.05 and ^+++^*p* < 0.001

**Fig. 5:**
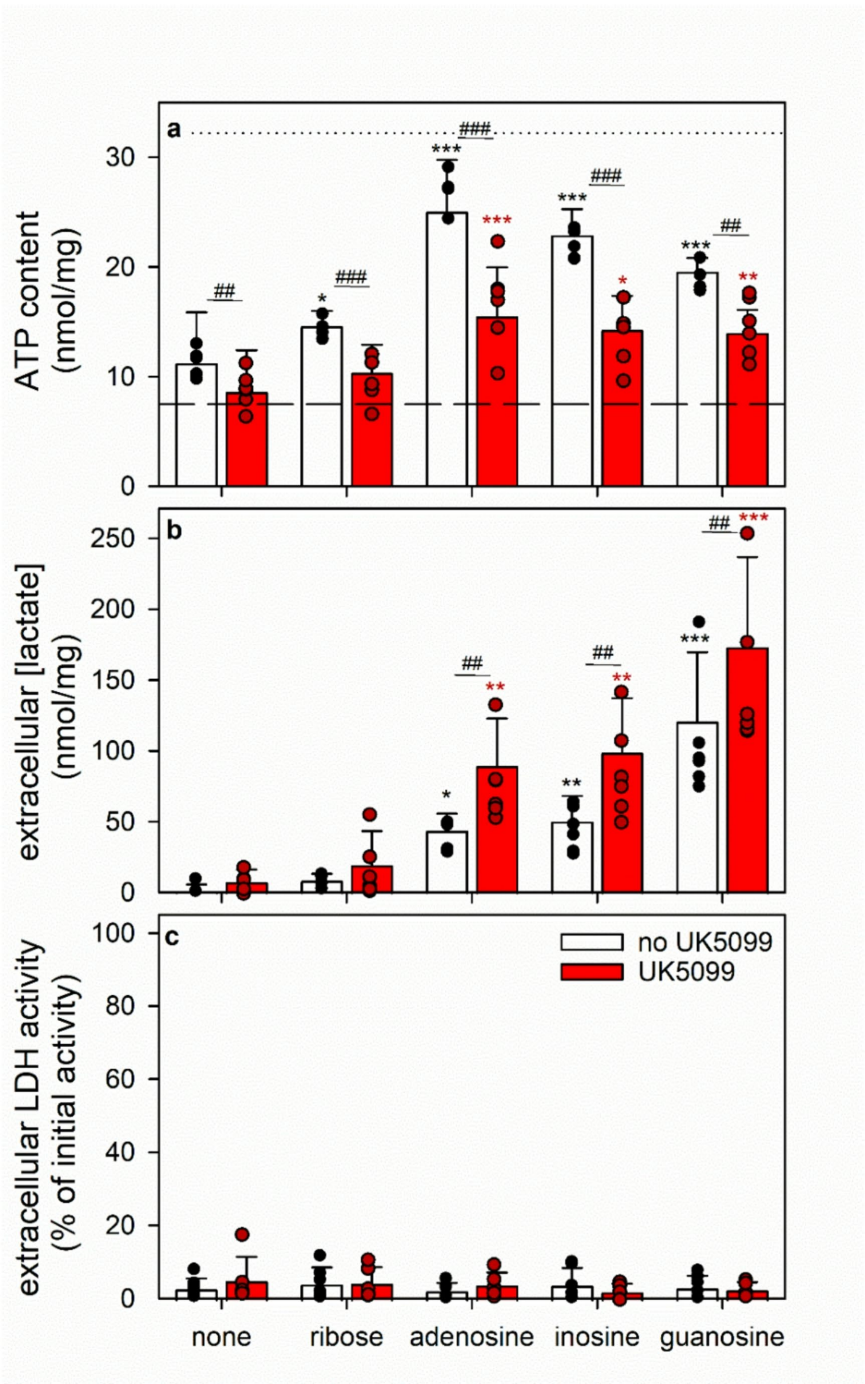
Use of ribose and purine nucleosides for ATP restoration and lactate formation in ATP-deprived astrocytes. The cultures had been pre-incubated for 60 min in glucose-free IB with 1 µM of BAM15 to lower the cellular ATP content before the cells were incubated in the absence or the presence of 1 mM of the given substrates with or without the mitochondrial pyruvate carrier inhibitor UK5099 (10 µM). The cellular ATP contents (**a**), the extracellular lactate accumulations (**b**) and the extracellular LDH activities (**c**) were determined after 60 min of incubation. The extracellular lactate concentrations of astrocytes that had been incubated with glucose as substrate for 60 min were 1036 ± 163 nmol/mg (control) and 1233 ± 189 nmol/mg (presence of UK5099). In panel a, the initial ATP content of the cultures and the ATP content determined after the 60 min preincubation (Table 1) are indicated by the black dotted line and the dashed line, respectively. The significance of differences (ANOVA; *n* = 6) compared with the data obtained for the respective control incubation (none) is indicated by *p < 0.05, **p < 0.01 and ***p < 0.001 in the colours of the respective bars. The significance of differences (t-test; *n* = 6) between data obtained for incubations with or without UK5099 is indicated by ^#^*p* < 0.05, ^##^*p* < 0.01 and ^###^*p* < 0.001

**Fig. 6:**
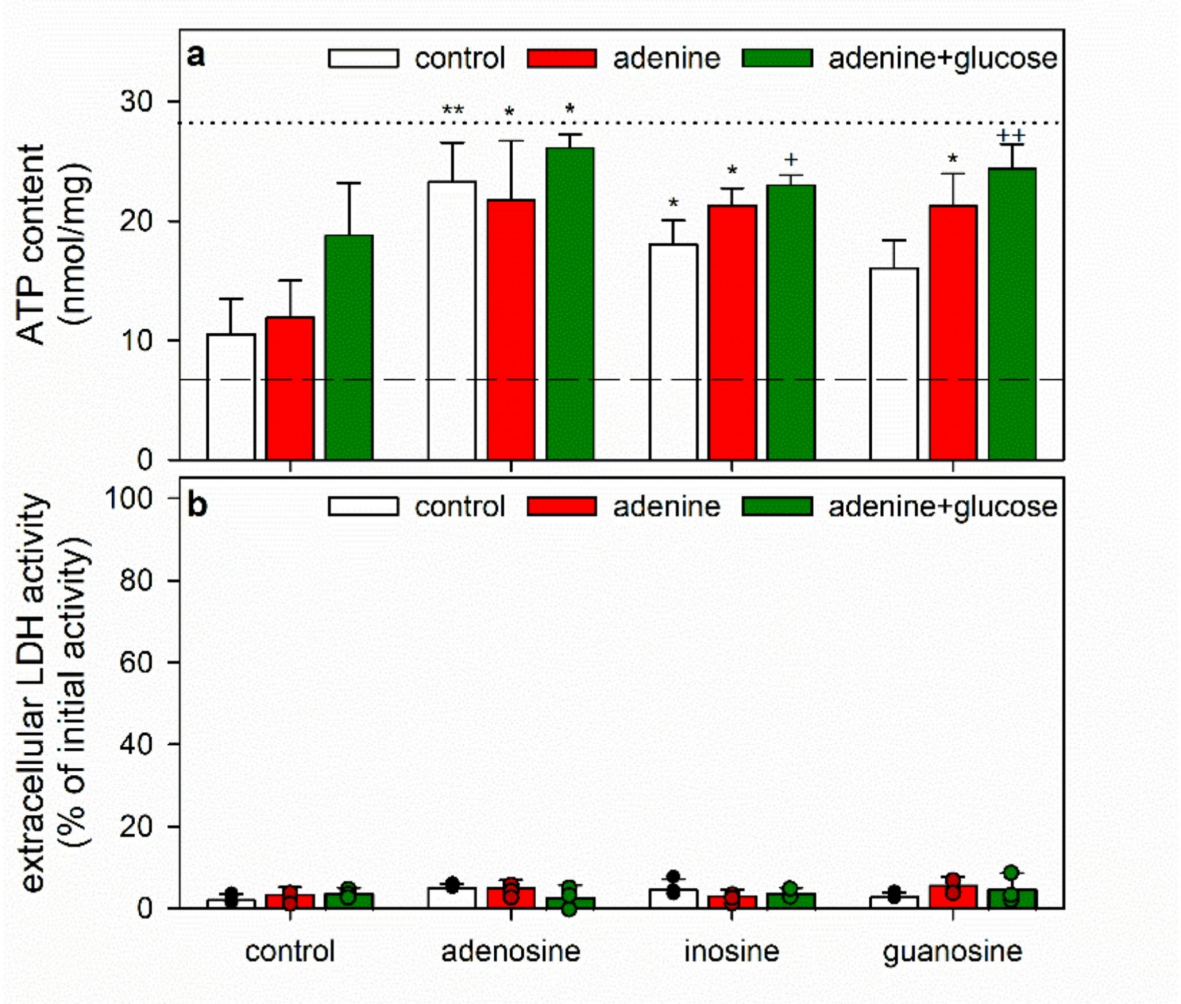
ATP restoration by ATP-deprived astrocytes in the absence or the presence of purine nucleosides, adenine and glucose. The cells had been preincubated for 60 min in glucose-free IB with 1 µM of BAM15 to lower the cellular ATP content before the cells were incubated without (none) or with 1 mM of the given nucleosides in the absence (control) or the presence of 1 mM adenine without or with 5 mM glucose for 60 min before the cellular ATP contents (**a**) and the extracellular LDH activities (**b**) were determined. In panel a, the initial ATP content of the cultures and the ATP content determined after the 60 min preincubation (Table 1) are indicated by the black dotted line and the dashed line, respectively. The significance of differences (ANOVA; *n* = 3) compared with the data obtained for the respective nucleoside-free control incubation (none) is indicated by *p < 0.05 and **p < 0.01. The significance of differences (ANOVA; *n* = 3) compared with the ATP data obtained for the respective incubations without adenine and glucose (control) is indicated by ^+^*p* < 0.05 and ^++^*p* < 0.01

**Fig. 7:**
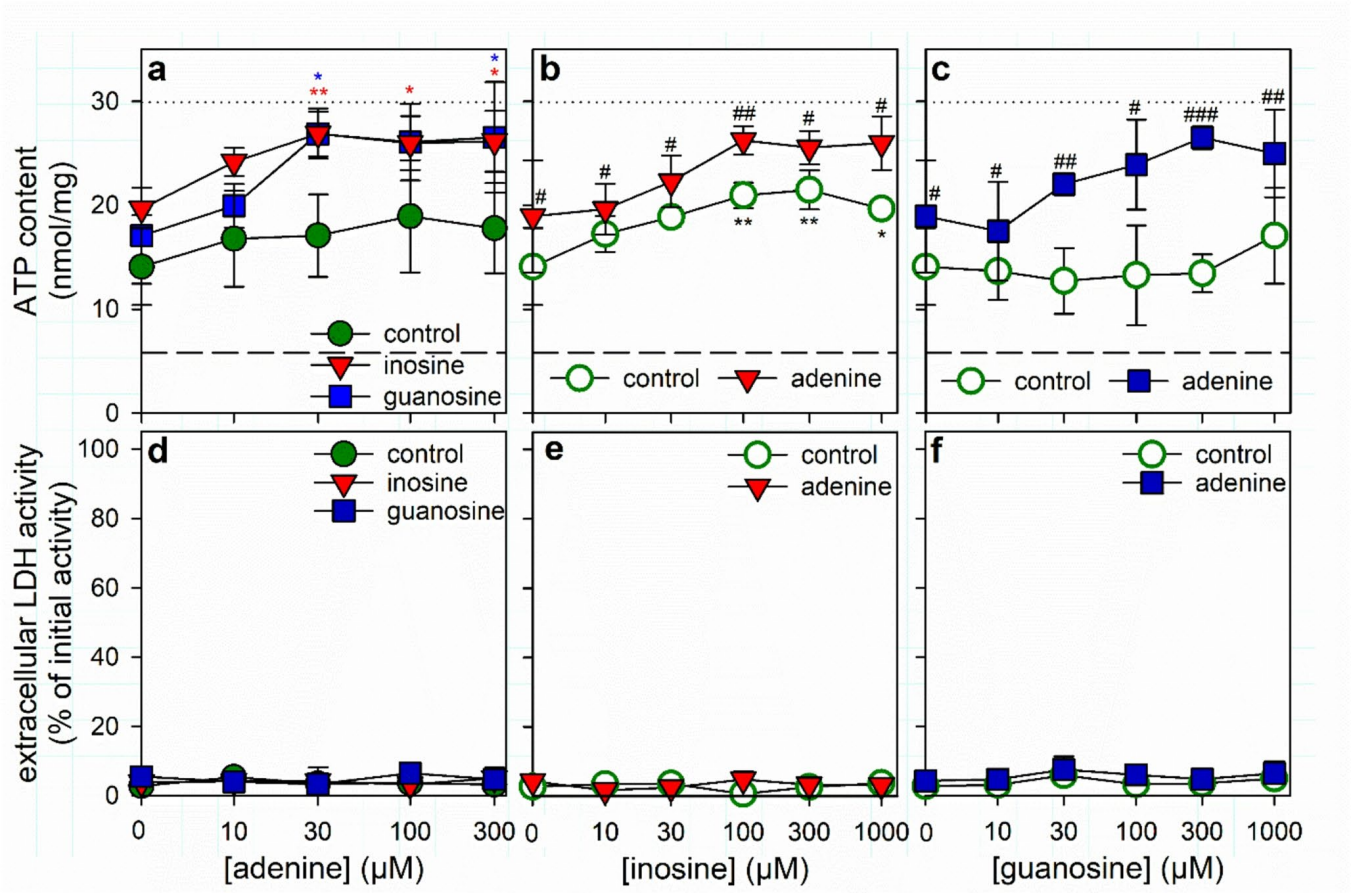
Dependency of the ATP restoration by ATP-deprived astrocytes on the concentrations of adenine, inosine or guanosine. The cells had been preincubated for 60 min in glucose-free IB with 1 µM of BAM15 to lower the cellular ATP content before the cells were incubated in glucose (5 mM)-containing buffer either without (control) or with 1 mM of inosine or guanosine in the presence of the given concentrations of adenine (**a**, **d**) or without (control) or with 100 µM adenine in the presence of the given concentrations of inosine (**b**, **e**) or guanosine (**c**, **f**). After 60 min of incubation, the cellular ATP contents (**a–c**) and the extracellular LDH activities (**d–f**) were determined. In panels a-c, the initial ATP content of the cultures and the ATP content determined after the 60 min preincubation (Table 1) are indicated by the black dotted lines and the dashed lines, respectively. In panel a and d, the significance of differences (ANOVA; *n* = 3) compared with the data obtained for the nucleoside-free control incubation (control) is indicated by *p < 0.05 and **p < 0.01 in the colors used for the symbols representing the indicated nucleosides. In panels b, c, e and f, the significance of differences (ANOVA; *n* = 3) compared with the data obtained for the respective nucleoside-free control incubation (0 µM) is indicated by *p < 0.05 and **p < 0.01. In these panels, the significance of differences (t-test; *n* = 3) between data obtained for incubations with or without adenine is given by ^#^*p* < 0.05, ^##^*p* < 0.01 and ^###^*p* < 0.001

**Fig. 8:**
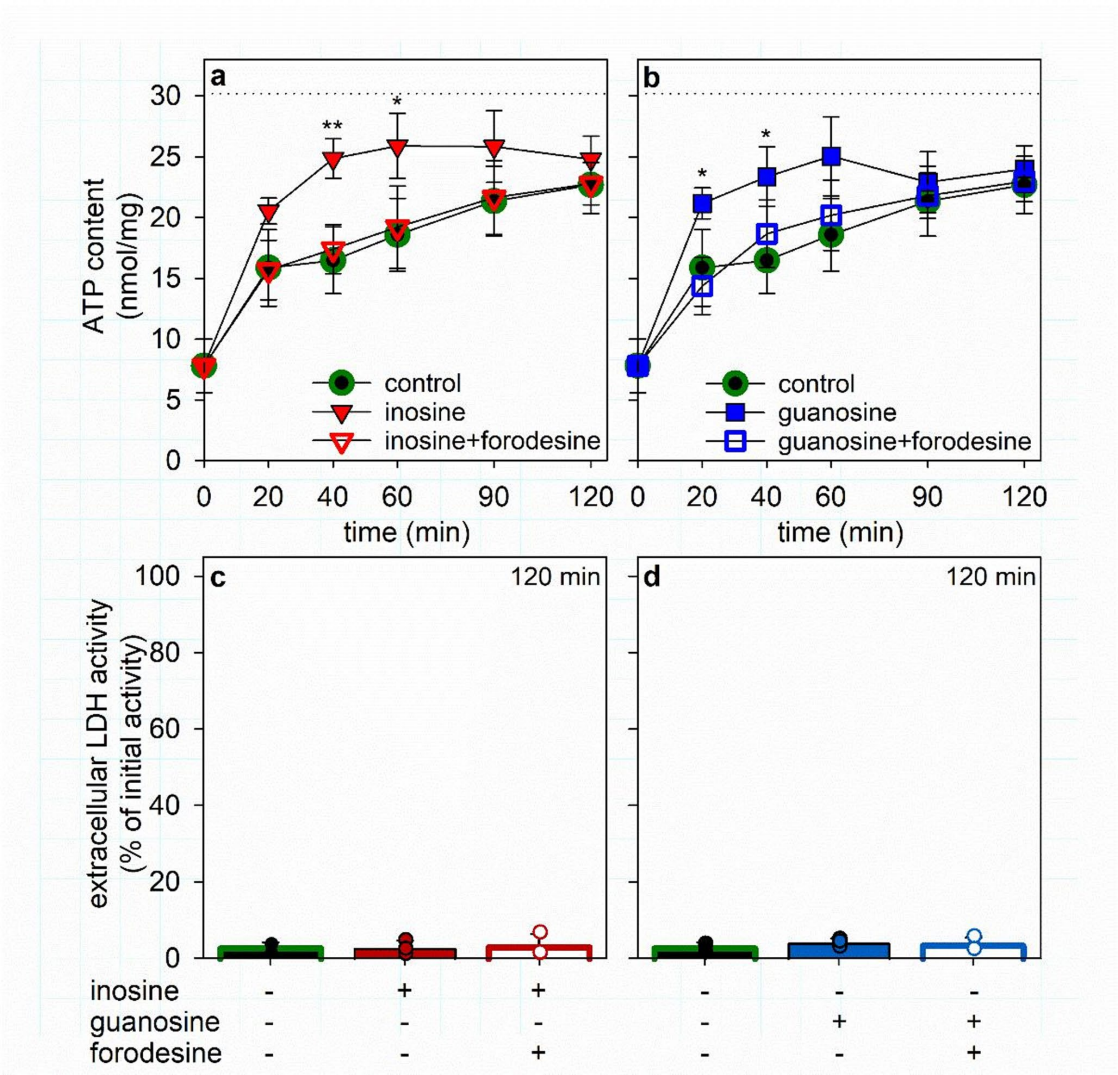
Time-dependency of the ATP restoration by ATP-deprived cultured astrocytes in the presence of adenine and purine nucleosides. The cells had been preincubated for 60 min in glucose-free IB with 1 µM of BAM15 to lower the cellular ATP content before the cells were incubated in glucose (5 mM)-containing buffer with 30 µM adenine (control) in the absence or the presence of either 300 µM inosine (**a**) or 300 µM guanosine (**b**). Some of the incubation media contained additionally the PNP inhibitor forodesine (10 µM). The cellular ATP contents (**a**, **b**) were determined for the given incubation periods and the extracellular LDH activities for the 120 min time point (**c**). In panels a and b, the initial specific ATP content (Table 1) is indicated by the dotted lines. The significance of differences (ANOVA; *n* = 3) of data compared to the values determined for the control condition is indicated by *p < 0.05 and **p < 0.01

Following this preincubation, ATP restoration was investigated for incubations of up to 6 h in incubation buffers containing the given substrates and/or enzyme inhibitors. For all experimental conditions used for this study, a potential loss in cell viability was investigated by measuring the extracellular LDH activity as indicator for damaged cell membranes. The determined extracellular LDH activities are given in all figures. None of the conditions investigated caused any significant increase in the extracellular LDH activity compared with the respective control condition (Figs. 1, 2, 3, 4, 5, 6, 7 and 8) consistent with literature data [17], demonstrating that the inability of astrocytes to restore ATP under a given condition was not a consequences of impaired membrane integrity.

To test for the potential of astrocytes to use free purine bases and/or purine nucleosides as substrates for ATP restoration, BAM15-treated astrocytes were incubated without or with 1 mM of adenine, hypoxanthine, adenosine, inosine or guanosine in the presence or the absence of 5 mM glucose for up to 6 h (Fig. 1). In the absence of any substrates, cellular ATP levels declined further during 6 h of incubation (Fig. 1a), while the presence of glucose as exclusive substrate enabled the cells to restore ATP by around 70% during the initial 2 h of incubation (Fig. 1a). Longer incubations did not lead to a substantial further increase in cellular ATP content (Fig. 1a). The data obtained for incubations in the presence of adenine (Fig. 1c) or hypoxanthine (Fig. 1e) did not differ significantly to those obtained for treatments without the bases (Fig. 1a). In contrast, the presence of adenosine (Fig. 1b), inosine (Fig. 1d) or guanosine (Fig. 1f) allowed in the absence of glucose substantial ATP restoration during the initial phase of the incubation that reached after around 120 min maximal values accounting for around 90%, 70% and 70% of the initial cellular ATP content, respectively. The presence of glucose plus guanosine (Fig. 1f) did not elevate cellular ATP contents above the level that was found for astrocytes that had been treated with glucose as exclusive substrate in the absence of nucleosides (Fig. 1a). For the co-application of glucose plus adenosine a rapid and almost complete ATP restoration was found (Fig. 1b), while co-application of inosine with glucose (Fig. 1d) accelerated the initial ATP restoration during the first 60 min compared to the glucose-only condition (Fig. 1a), but did not lead to a further increase in cellular ATP content during longer incubation (Fig. 1d).

Investigation of the concentration-dependencies of the initial ATP restoration (60 min) in the presence of purine nucleosides revealed concentration-dependent increases in ATP restoration both in the absence and the presence of glucose for adenosine (Fig. 2a) and inosine (Fig. 2b), but not for incubations with guanosine (Fig. 2c). For all conditions investigated, the presence of glucose improved the initial ATP restoration found for incubations with the purine nucleosides (Fig. 2).

For further experiments that studied ATP restoration in the absence of glucose, the purine nucleosides were applied in a high concentration of 1 mM to provide sufficient substrate to enable the cells to use the nucleosides both as energy source and as potential supplier of components for the synthesis of new AMP. For most of the ATP restoration experiments that were performed in the presence of glucose as energy substrates, micromolar concentrations of nucleosides and/or purines were applied as such lower concentrations were found to be sufficient to supply essential components for AMP synthesis and ATP restoration in the treated astrocytes.

### Test for a Contribution of PNP in the ATP Restoration from Nucleosides in ATP-deprived Astrocytes

To test for the involvement of purine nucleoside phosphorylase (PNP) in the utilization of purine nucleosides as substrates from ATP restoration, the PNP inhibitor forodesine [49, 50] was applied. During incubation for 60 min in the absence of glucose, forodesine lowered significantly the ATP restoration in ATP-deprived astrocytes that had been exposed to 1 mM adenosine, inosine or guanosine (Fig. 3a). In contrast, in glucose-fed astrocytes the ATP restoration found for incubations with 30 µM inosine, but not for that with adenosine, was significantly lowered by forodesine, while guanosine was unable to improve ATP restoration in glucose-fed astrocytes (Fig. 3b).

### Test for Ribose as a Potential Substrate for ATP Restoration in ATP-deprived Astrocytes

Adenosine is efficiently used by astrocytes both as AMP precursor and energy substrate for ATP restoration (Fig. 1; [17]). To test whether a combination of the two adenosine components, adenine and ribose, or other combinations of purine bases and nucleosides with ribose may foster ATP restoration, BAM15-treated astrocytes were incubated without a sugar, with ribose or with glucose in the absence or the presence of 1 mM adenine, hypoxanthine, adenosine, inosine or guanosine for 60 or 360 min (Fig. 4). The results obtained for incubations without or with glucose were consistent with those found previously for the respective conditions (Fig. 1). In contrast, significant ATP restoration was not observed with any of the ribose-containing treatments compared to the respective sugar-free control incubation (Fig. 4), showing that extracellular ribose is not fueling ATP restoration under the conditions used.

### Test for the Potential Utilization of Ribose and Purine Nucleosides as Energy Substrate for ATP Restoration in ATP-deprived Astrocytes

Purine nucleosides have been shown to serve as exogenous substrates that fuel the maintenance of a high ATP content in glucose-deprived astrocytes [16]. To test to which extent astrocytes metabolize nucleosides or ribose to lactate via the PPP and glycolysis, BAM15-treated astrocytes were incubated for 60 min in glucose-free buffer with 1 mM of ribose, adenosine, inosine or guanosine in the absence or the presence of UK5099, an inhibitor of the mitochondrial pyruvate carrier [51, 52]. While at best little ATP restoration was observed for ribose-treated astrocytes, substantial amounts of ATP had been restored in the presence of adenosine, inosine or guanosine (Fig. 5a) consistent with previously shown data (Figs. 1b, d and f and 3a). The presence of UK5099 significantly lowered the ATP restoration by approximately 50% compared to the respective inhibitor-free treatment (Fig. 5a), supporting published data that mitochondrial ATP regeneration is needed for efficient ATP restoration at least from adenosine in cultured astrocytes [17].

Lactate generation and release was investigated as second indicator for the metabolism of exogenous ribose or purine nucleosides by astrocytes. Lactate release was hardly detectable for ATP-deprived astrocytes that had been incubated without substrate or with ribose (Fig. 5b). In contrast, for incubations of astrocytes with each of the three nucleosides substantial lactate production and release was observed. Guanosine-treated astrocytes produced more lactate than cells that had been exposed to 1 mM adenosine or inosine (Fig. 5b). However, the amounts of lactate released by nucleoside-treated astrocytes was one order of magnitude lower than that found for glucose-treated astrocytes under identical incubation conditions (1036 ± 163 nmol/mg). For all nucleosides, the amount of lactate found released from the cells was significantly increased in the presence of UK5099 (Fig. 5b). Among the different purine nucleosides, guanosine is the best exogenous substrate for ATP-deprived astrocytes to fuel carbon into the lactate production via PPP and glycolysis.

### Test of Combinations of Exogenous Precursors To Enable Rapid ATP Restoration in ATP-deprived Astrocytes

The combination of adenosine with an energy substrate has been reported to enable efficient and rapid ATP restoration in ATP-deprived astrocytes [17]. In this combination, adenosine supplies both the adenine and ribose moiety for the synthesis of new AMP, while glucose is used as energy substrate. In order to test for combinations of exogenous precursors as potential replacement for the neuroactive adenosine, mixtures of adenine with the purine nucleosides inosine and guanosine were tested for their potential to enable rapid ATP restoration in ATP-depleted astrocytes within 60 min (Fig. 6).

In the absence of nucleosides, only a marginal cellular ATP restoration was found that was not improved by the presence of adenine, while application of adenine plus glucose enabled the cells to restore some ATP (Fig. 6a), consistent with data shown in Fig. 4a. Incubations with adenosine as positive control for ATP restoration [17] enable almost complete ATP restoration that was not further improved by the additional presence of adenine or adenine plus glucose (Fig. 6a). In contrast, for incubations with either inosine or guanosine the partial ATP restoration found of nucleoside-only treatments were increased in the presence of adenine plus glucose (Fig. 6a). After 60 min incubation of glucose-fed astrocytes with adenine plus inosine or guanosine the ATP contents restored were almost identical to those found for incubation with adenosine-containing medium, representing around 80–90% of the initial ATP content (Fig. 6a). These data demonstrate that a combination of adenine plus either inosine or guanosine can foster rapid ATP restoration in glucose-fed astrocytes.

Investigations of the concentration-dependencies for adenine (Fig. 7a), inosine (Fig. 7b) and guanosine (Fig. 7c) revealed that in glucose-fed astrocytes already the presence of 30 µM adenine (in the presence of 1 mM nucleosides) (Fig. 7a) fostered maximal ATP restoration within 60 min, while in the presence of 100 µM adenine maximal ATP restoration were found for 100 µM inosine (Fig. 7b) and 300 µM guanosine (Fig. 7c).

The data shown in Fig. 7 revealed that in glucose-fed astrocytes already 30 µM adenine (Fig. 7a) and 300 µM inosine or guanosine (Fig. 7b, c) were sufficient to foster efficient ATP restoration in ATP-deprived astrocytes. Therefore, these concentrations of adenine and of the purine nucleosides were now applied for ATP restoration in coincubation experiments. Application of 30 µM adenine with glucose enabled ATP-deprived astrocytes to restore within 120 min around 75% of the initial ATP content (Fig. 8a, b). However, if 300 µM inosine (Fig. 8a) or guanosine (Fig. 8b) were applied together with adenine and glucose, the ATP restoration was strongly accelerated and maximal ATP restoration was found already after 40 min of incubation, accounting for around 80% of the initial cellular ATP content (Fig. 8a, b). The supporting effect of the purine nucleosides on the ATP restoration in the presence of adenine plus glucose was completely abolished in the presence of the PNP inhibitor forodesine (Fig. 8a, b), demonstrating that metabolism of the two nucleosides by PNP is involved in their stimulating effects on ATP restoration in ATP-depleted astrocytes.

## Discussion

The depletion of ATP in astrocytes is accompanied by a loss in ADP and AMP [14, 17]. Therefore, the cells have to synthesize new AMP as building block for restoration of their initial high cellular ATP content. We have recently shown that ATP depleted astrocytes restore ATP slowly in the presence of glucose as exclusive substrate, while cellular ATP restoration is strongly accelerated by the application of adenosine in a process that involves phosphorylation of adenosine by adenosine kinase [17]. However, adenosine has multiple functions as extracellular signaling molecule in astrocytes and other brain cell types [42, 53] and is therefore not suitable as potential substrate to improve ATP restoration in brain [24]. We have now extended our initial study [17] by testing various compounds as well as their combinations for the potential to replace adenosine as extracellular substrate to foster rapid ATP restoration in ATP-depleted astrocytes by making use of the purine salvage pathway. Brain cells are considered to use mainly the purine salvage pathway and not the *de novo* synthesis of IMP for the synthesis of AMP [24, 25]. For the conditions of our studies, even residual *de novo* purine synthesis to IMP and subsequent amidation to AMP is unlikely to contribute substantially to the ATP restoration as the basal incubation buffer does not contain the substrates needed for the *de novo* synthesis.

The use of adenosine for ATP restoration in the presence of glucose is completely prevented by inhibition of adenosine kinase, while in the absence of glucose ATP restoration is prevented by inhibition of mitochondrial pyruvate uptake or oxidative phosphorylation [17]. These data suggest that adenosine can serve both as building block (substrate for adenosine kinase) and also as energy substrate (use of the ribose moiety of adenosine for ATP regeneration via PPP, glycolysis and oxidative phosphorylation) for ATP restoration in ATP-depleted astrocytes [17]. Accordingly, we used in many experiments a treatment with adenosine as positive control for ATP restoration. All results observed in the current study for incubations of astrocytes with adenosine are consistent with those previously reported [17]. In contrast to adenosine, the presence of the purine bases adenine or hypoxanthine did at best allow a slow acceleration of the ATP restoration in the presence of glucose. Also, an application of ribose as potential precursor of the PRPP needed by PRT as substrate to synthesize with a purine base the respective nucleoside monophosphate was not suitable to accelerate ATP restoration in the absence of glucose, neither in the presence of purine bases nor in the presence of purine nucleosides. This contrasts results from ATP restoration experiments on brain slices that reported ATP restoration for treatments with the combination of ribose plus adenine [24, 54]. For cultured astrocytes, extracellular ribose appears to be a rather poor substrate as also shown by the at best marginal amounts of lactate that were found in the media of ribose-treated astrocytes. Ribose is most likely taken up into mammalian cells by members of the GLUT transporter family [55] and is phosphorylated in cells by ribokinase to ribose-5-phosphate [56]. For astrocytes, most likely the phosphorylation is limiting the cellular ribose utilization as the expression level of ribokinase appears to be rather low in astrocytes [57, 58]. Nevertheless, further studies are required to elucidate whether astrocytes are able to take up and phosphorylate ribose and to explore under which conditions extracellular ribose may serve as substrate for the astrocytic metabolism.

Partial ATP restoration was found for ATP-depleted astrocytes that had been exposed to the purine nucleosides inosine or guanosine. Similar to adenosine, also inosine and guanosine are taken up into cells by equilibrative nucleoside transporters [30–36] that are expressed in astrocytes [25, 37, 38]. After uptake into cells, the metabolism of purine nucleosides involves phosphorolytical cleavage to ribose-1-phosphate and free purine by PNP [21, 25, 39]. This enzyme accepts adenosine, inosine and guanosine as substrates with similar K_M_ values [59–63]. The nucleoside-derived ribose-1-phosphate can be converted by phosphoribose isomerase [39, 64] to ribose-5-phosphate that is channeled either via the PPP into glycolysis or is used to synthesize PRPP.

Cultured astrocytes are able to take up and metabolize both inosine and guanosine as demonstrated by the ability of these nucleosides to partially restore ATP in the absence of glucose, by the ability of the PNP inhibitor forodesine to lower the use of both nucleosides as energy substrate for ATP restoration, and by the formation and release of lactate during incubation of astrocytes with inosine or guanosine as exclusive substrates. This lactate formation was even accelerated in the presence of the mitochondrial pyruvate uptake inhibitor UK5099, as previously reported for adenosine-treated astrocytes [17], showing that a substantial part of the nucleoside-derived pyruvate is oxidized in mitochondria. Interestingly, cultured astrocytes were able to produce even more lactate from guanosine than from adenosine or inosine, demonstrating a high potential of astrocytes to metabolize guanosine. The use of exogenous purine nucleosides by astrocytes as energy substrate is consistent with literature data showing that these substances help to maintain a high cellular ATP content in starved astrocytes [16, 40].

Among the investigated purine nucleosides, adenosine was the most potent to accelerate ATP restoration, while incubation with inosine or guanosine led only to partial ATP restoration in ATP-depleted astrocytes. Of these two purine nucleosides, inosine appears to be more potent to support ATP restoration as maximal ATP contents were restored earlier in the presence of inosine than with guanosine and at lower nucleoside concentrations. While the presence of inosine enabled astrocytes to restore ATP to a level that was exceeding the content observed for incubations with glucose alone, the use of exogenous guanosine was unable to do so. Inosine metabolism can lead to AMP formation via PNP-mediated liberation of hypoxanthine that is connected with PRPP by hypoxanthine-guanine PRT to IMP that is finally amidated to AMP [25]. In contrast, astrocytes are unable to use guanosine to generate an adenine-containing nucleoside or nucleotide that may serve as building block for ATP restoration under the conditions studied.

Coincubations of ATP-depleted astrocytes with adenine in the presence of glucose did slightly accelerate ATP restoration above the level found for glucose-only treated astrocytes. This suggests that either the uptake or intracellular utilization of adenine as substrate of adenine PRT or the generation of PRPP from glucose limits the synthesis of new AMP that is subsequently used for ATP restoration. As ribose failed to enable ATP restoration in the presence of adenine, we explored whether extracellular inosine or guanosine may serve as precursor to generate efficiently the PRPP that is needed for AMP synthesis in the presence of coapplied adenine. Indeed, the addition of inosine or guanosine already in micromolar concentrations strongly accelerated ATP restoration in astrocytes incubated with adenine plus glucose to rates that were similar to those found for adenosine-treated astrocytes. This suggests that insufficient supply of PRPP in glucose-fed astrocytes may slow ATP restoration in the presence of adenine and that the application of inosine or guanosine can bypass this limitation by providing additional ribose phosphate for the synthesis of PRPP. This view is strongly supported by the observation that the stimulation of ATP restoration by inosine and guanosine was completely prevented by inhibition of PNP, the enzyme generating ribose-1-phosphate from the nucleosides.

In conclusion, efficient and rapid ATP restoration in ATP-deprived astrocytes was observed for treatments that combine three extracellular substrates, glucose as energy substrate, adenine as precursor for the adenine moiety of ATP, and inosine or guanosine as extracellular precursor for the ribose phosphate moiety of ATP. Such combinations should now also be tested for their potential to maintain or even increase ATP contents in other types of brain cells under normal and stressed conditions.

In brain, rapid ATP loss is a consequence of hypoxic and ischemic conditions [65–69] and traumatic mechanical impacts [24, 70, 71], although it is currently unclear whether and to which extent a lowered ATP content in brain astrocytes may contribute to the overall ATP loss observed. However, as astrocytes are considered to play a major role in controlling the equilibrium between adenosine phosphates and adenosine in brain [53] and as an unbalanced energy metabolism in astrocytes has been connected with brain pathology [72, 73], it is worthwhile to further explore strategies to help astrocytes to maintain or restore their ATP in vivo. Both inosine and guanosine differ substantially in their neuroactive potential compared to adenosine and have even been considered as beneficial compounds for treatments of brain malfunction [30, 74–76]. Thus, combinations of inosine or guanosine as extracellular PRPP precursors with adenine may also be suitable to improve ATP restoration in the hypoxic or traumatized brain.

## Data Availability

Enquieries on original data should be directed to the corresponding author.
